# VCF2PCACluster: a simple, fast and memory-efficient tool for principal component analysis of tens of millions of SNPs

**DOI:** 10.1186/s12859-024-05770-1

**Published:** 2024-05-01

**Authors:** Weiming He, Lian Xu, JingXian Wang, Zhen Yue, Yi Jing, Shuaishuai Tai, Jian Yang, Xiaodong Fang

**Affiliations:** 1 BGI Research, Sanya, 572025 People’s Republic of China; 2 Key Laboratory of Neuroregeneration of Jiangsu and Ministry of Education, Co-innovation Center of Neuroregeneration, NMPA Key Laboratory for Research and Evaluation of Tissue Engineering Technology Products, Nantong University, Nantong, 226001 People’s Republic of China

**Keywords:** VCF2PCACluster, Principal component analysis (PCA), Clustering, Visualization, SNP, VCF

## Abstract

Principal component analysis (PCA) is an important and widely used unsupervised learning method that determines population structure based on genetic variation. Genome sequencing of thousands of individuals usually generate tens of millions of SNPs, making it challenging for PCA analysis and interpretation. Here we present VCF2PCACluster, a simple, fast and memory-efficient tool for Kinship estimation, PCA and clustering analysis, and visualization based on VCF formatted SNPs. We implemented five Kinship estimation methods and three clustering methods for its users to choose from. Moreover, unlike other PCA tools, VCF2PCACluster possesses a clustering function based on PCA result, which enabling users to automatically and clearly know about population structure. We demonstrated the same accuracy but a higher performance of this tool in performing PCA analysis on tens of millions of SNPs compared to another popular PLINK2 software, especially in peak memory usage that is independent of the number of SNPs in VCF2PCACluster.

## Introduction

With the advancement of the next-generation sequencing techniques and the decrease in cost, it is now feasible to perform large-scale genome sequencing of thousands of individuals by large consortiums or even labs, generating large-scale genotype data containing tens of millions of single-nucleotide polymorphisms (SNPs), such as the 1000 Genome Project, UK Biobank and 3000 Rice Genomes Project [1–3]. Principal component analysis (PCA) has been widely used in the study of population genetics for many years [4]. Existing popular tools for implementing such analysis include toolkits such as TASSEL, GAPIT, PLINK2 and GCTA [5–8]. However, these tools may not be suitable for the analysis of billion-level SNPs due to a large computational resource consumption and some of them require format conversion and/or multiple steps to finish. Moreover, advanced analysis based on the PCA result for large-scale samples, such as clustering and visualization, is also needed. To this end, we developed a dedicated and user-friendly PCA analysis tool, VCF2PCACluster. This tool can easily calculate Kinship matrix and perform PCA and clustering analysis, and yield publication-ready 2D and 3D plots based on the variant call format (VCF) formatted SNP data in a fast and low-memory usage. Specifically, the memory usage of this tool is independent of the number of SNP sites and running time is as fast as the performance of PLINK2 and GCTA, making it more applicable in a large-scale genome-wide study with tens of millions of SNPs.

## Implementation

**Fig. 1:**
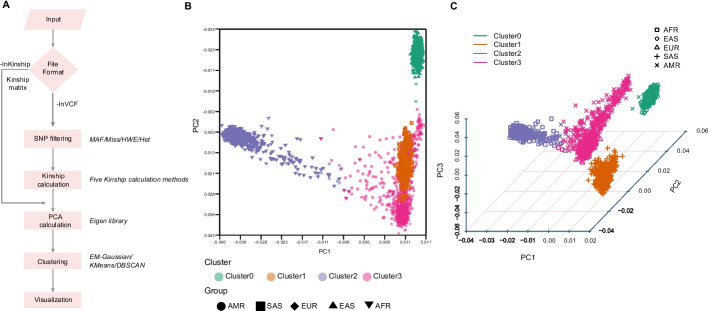
Overview of the VCF2PCACluster and PCA visualization of sample clusters. **A** Five major modules implemented in the VCF2PCACluster, SNP filtering, Kinship estimation, PCA analysis, Clustering and Visualization. The VCF formatted data (-InVCF) or Kinship matrix (-InKinship) could be adopted by the VCF2PCACluster. **B** and **C** Visualization of PCA and clustering result in 2D and 3D plots on human Chr22 SNP data from 1000 Genome Project. Colors indicate different clusters determined by VCF2PCACluster. Shapes indicate prior knowledge of geographic population (*AFR* African Region, *AMR* Region of the Americas, *EUR* European Region, *EAS* East Asian and *SAS* South Asian)

VCF2PCACluster, a command-line tool, is implemented using programming languages C++ and Perl. The C++ was used for the calculation process and Perl was used for visualization. This tool enables its users to provide only the general VCF formatted input, and rapidly and directly yields the results of Kinship matrix, PCA, clustering and visualization in publication-ready 2D and 3D plots. It consists five major steps (Fig. 1) and is briefly described as followings.

Genome sequencing in population study usually generates tens of millions of SNPs across thousands of samples, making the PCA analysis heavily depend on the extensive computer resource, such as CPU and memory. Despite the efforts of PLINK2 and GATC to optimize memory usage with the use of two bits to store genotype, the PCA analysis of thousands of samples and billions of SNPs still requires hundreds of gigabytes (GB). To address this challenge, we have adopted a processing strategy that operates in a line-by-line manner, which involves reading a site (line) and subsequently calculating the difference between pairwise samples. The results are stored in two arrays ([sample × sample]) for final calculation of the kinship matrix. Consequently, the memory usage during the execution is influenced solely by the sample size, rather than the number of SNPs. For CPU running time, it is limited by both the number of samples and SNPs. To improve efficiency, we employed a two-dimensional array to store frequently accessed data and utilized multi-threading through the OpenMP library [9] to calculate the Kinship matrix. By using approaches, VCF2PCACluster is capable of handling a large-scale SNP data with fast processing speed and minimal memory consumption.

Due to the imperfect of raw genotype data, obtaining a high-quality of SNP data is the first key step to generate reliable results in population genetic studies. Typic filtering criteria include a certain ratio of SNP missingness, low call rate, *Hardy–Weinberg equilibrium* (HWE) and *low minor allele frequency* (MAF) [10]. For SNP filtering, non-biallelic sites (singleton and multiallelic) and indels will be discarded. Additionally, users may optionally exclude sites with low minor allele frequency (MAF), missingness per marker (missing) and concordance with Hardy–Weinberg equilibrium (HWE) by using parameters (*e.g.*, -MAF 0.05 -Miss 0.25 -HWE 0) based on VCF input format.

For Kinship estimation, we have implemented five typical methods: Normalized_IBS, Centerred_IBS [11], IBSKinshipImpute, IBSKinship [12] and *p* distance [13]. Normalized_IBS and Centered_IBS could improve PCA by considering genetic relatedness and mitigating confounding factors such as scale differences and population structure, resulting in enhanced precision, stability, and interpretability of the PCA analysis. Thus, we recommend that the first two kinship methods should be priority to applications. For PCA analysis, VCF2PCACluster utilizes the external eigen library [14] to compute PCA and eigenvalues based on either the previously calculated kinship matrix or one provided by the users. In terms of clustering step, we have implemented three clustering methods: EM-Gaussian [15], K-Means [16] and DBSCAN [17] based on the top three principal components (PCs). By default, we initially detected the best K (defining the number of clusters) and centroid using K-Means for the initial bootstrap and then perform 1000 bootstraps to determine the best clusters using EM-Gaussian. Considering the requirement of adjusting clustering methods and parameters for the best clustering results applied in different scenarios, we also provided a parameter (-InKinship) for users to re-cluster directly from previously obtained kinship matrix result. Finally, we offer two custom Perl scripts for users to generate publication-ready 2D and/or 3D PCA and clustering plots.

In addition, VCF2PCACluster enables users to perform analysis on a subset of samples defined in the VCF input using the (-InSubSample) parameter. It also enables comparisons between the prior sample group labels with the unsupervised clustering result through the (-InSampleGroup) parameter. For more details, please refer to our manual deposited on GitHub.

## Application

To evaluate the accuracy and performance of VCF2PCACluster, we utilized the SNP data from the Chr22 of the 1000 Genome Project, which contains a total of 1,055,401 SNPs across 2,504 samples. The test was completed in approximately 7 min with 16 threads and the running memory usage was about 0.1 GB. We compared the PCA result with that generated by GCTA and PLINK2, and found that they were identical. Based on the SNP clustering results, four clusters were inferred using VCF2PCACluster. Top two principal components (PCs) and three PCs were used to visualize the clustering result respectively (Fig. 1B,C). We could observe obvious four distinct populations from African (AFR), Asian (EAS/SAS) and European (EUR) and Americas (AMR) from the top three PCs (Fig. 1C). The consistency between the clustering result and predefined groups was measured to be 0.995, indicating a high accuracy of VCF2PCACluster in distinguishing subpopulations.

We also compared VCF2PCACluster with other popular tools (PLINK2, GCTA, Tassel, Gapit3) using the same test data in terms of the input format, pre-processing, function, and performance (Table 1). Both Tassel and Gapit3 used the most peak memory usage (> 150 GB) and a large time consumption (> 400 min), indicating that they are not suitable for analyzing large-scale SNP data. Both VCF2PCACluster and PLINK2 could adopt the general VCF format as input and have a similar optional pre-processing for filtering SNPs with low MAF, a high ratio of missing, and HWE. They exhibit comparable time consumption, but VCF2PCACluster demonstrates the smallest running memory usage (~ 0.1 GB) and implements additional functional modules including clustering and visualization. Next, we conducted a test on a very large dataset with a combination of SNP data on chromosomes 1–22 from the 1000 Genome Project, totaling of 81.2 million (M) SNPs. The results showed extremely low memory usage (~ 0.1 GB) and successfully finished in about 610 min with 8 threads using VCF2PCACluster. Conversely, PLINK2 required a larger memory usage (> 200 GB) and failed to complete the job. In addition, we also tested on a large-scale SNP dataset in rice with 3 k samples and 29 M SNPs [18]. We demonstrated that VCF2PCACluster can efficiently produce results, requiring 181 min and only 0.1 GB memory. In comparison, PLINK2 took 100 min and consumed a massive 257 GB of memory. These cases highlight the memory efficiency of VCF2PCACluster, even when analyzing tens of millions of SNPs. Detailed comparisons in accuracy and efficiency between VCF2PCACluster and other tools were described in the manual deposited on GitHub.

**Table 1:** Comparison of the VCF2PCACluster with other tools

Software	Input	SNP filtering	Functions				Performance	
			Kinship	PCA	Clustering	Visualization	Memory	Time consumption
VCF2PCACluster	VCF	Maf, Missing, HWE	Yes	Yes	Yes	Yes	~ 0.1 GB	~ 7 min (16 threads)
GCTA	Plink2	Maf	Yes	Yes	No	No	~ 1.5 GB	~ 7 min (16 threads)
PLINK2	VCF	Maf, Missing, HWE	Yes	Yes	No	No	~ 1.5 GB	~ 2.47 min (16 threads)
TASSEL	VCF/hmp	Maf	Yes	Yes	No	No	> 180 GB	> 400 min
GAPIT	hmp	no	No	Yes	No	Yes	> 150 GB	> 400 min

## Discussion and conclusion

PCA is an important statistic method for exploring population structure. Among the existing tools that implement similar analyses, VCF2PCACluster shows the best performance, especially in terms of memory usage and the ability to quickly re-cluster based on the prior analyses. Next, we will further improve VCF2PCACluster by adopting additional input formats, such as PLINK2, and implementing more clustering algorithm for users to choose from. Since its initial release one year ago, four studies have used VCF2PCACluster (previously known as MingPCACluster) [19, 20] to conduct their analyses and visualize the results.

In summary, VCF2PCACluster is a very simple and user-friendly software that enables users to perform PCA and clustering analyses using general VCF formatted SNP data for all or a subset of samples. It also facilitates for easy and rapid adjustment of clustering results from previous runs or external Kinship result. Notably, it can efficiently handle large-scale SNP data in a fast and memory-efficient manner, making it a suitable PCA and clustering software even for moderately powered computers.

## Availability and requirements

Project name: VCF2PCACluster.

Project home page: https://github.com/hewm2008/VCF2PCACluster

Operation system(s): Linux or Mac OS.

Programming language: C/C +  + 

Other requirements: gcc-4.9 or higher with OpenMP.

License: MIT license.

Any restrictions to use by non-academics: None.

## Data Availability

The data sets generated and/or analyzed during the current study are available in the https://github.com/hewm2008/VCF2PCACluster/tree/main/example.
